# Correction to Patchy Amphiphilic Dendrimers Bind Adenovirus and Control Its Host Interactions and *in Vivo* Distribution

**DOI:** 10.1021/acsnano.0c10327

**Published:** 2021-01-06

**Authors:** Yuzhou Wu, Longjie Li, Larissa Frank, Jessica Wagner, Patrizia Andreozzi, Brenton Hammer, Marco D’Alicarnasso, Maria Pelliccia, Weina Liu, Sabyasachi Chakrabortty, Silke Krol, Johanna Simon, Katharina Landfester, Seah Ling Kuan, Francesco Stellacci, Klaus Müllen, Florian Kreppel, Tanja Weil

On page 8753, the blood vessel background is missing in the last picture of Figure 3d. The corrected Figure 3d is as follows:

**Figure 3:**
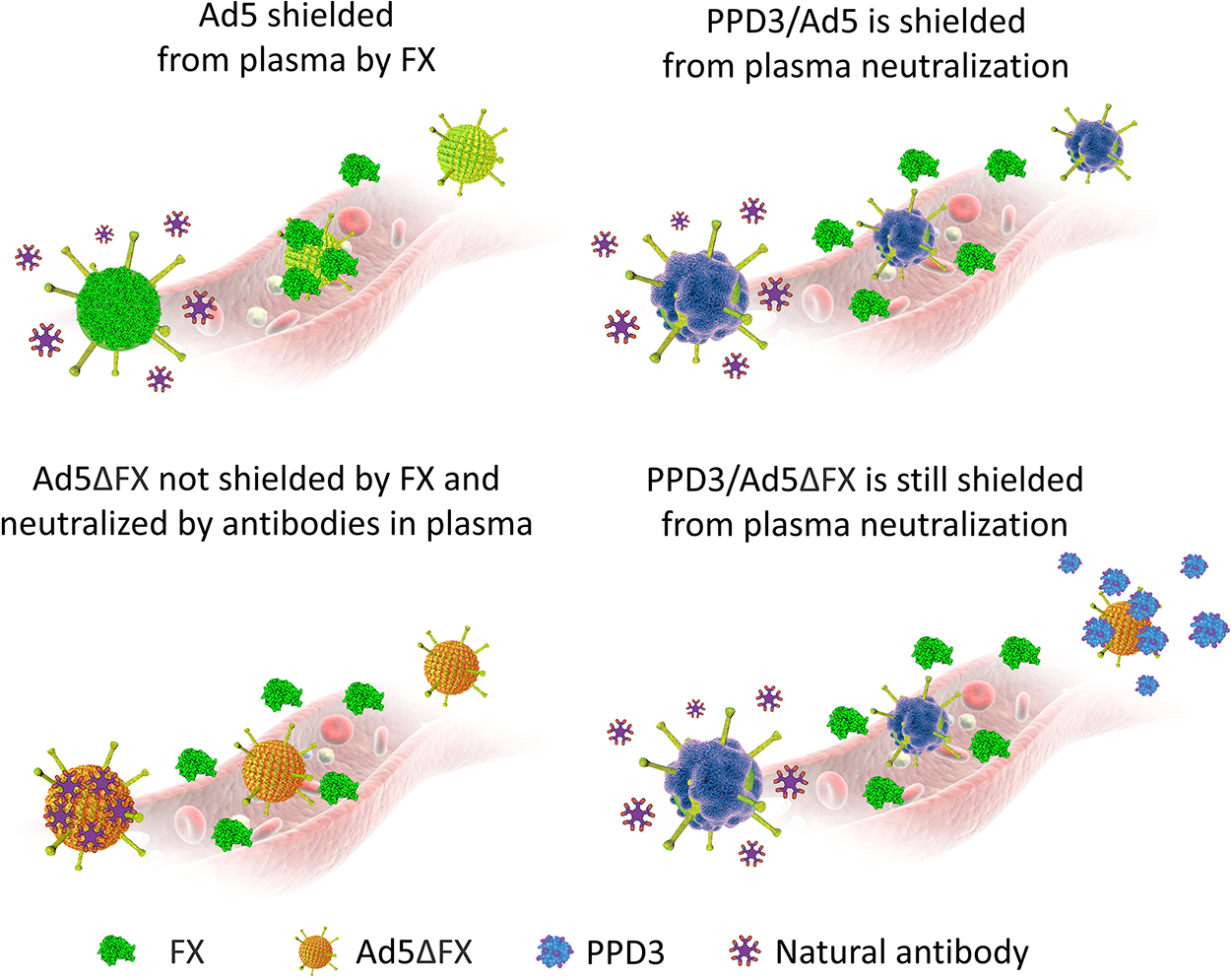
PPD3/Ad5 complexes exhibit increased transduction efficiency in human plasma and PPD3 protected FX-binding ablated capsids from neutralization by the IgM/complement pathway. (d) Comparison of uncoated Ad5, PPD3/Ad5, Ad5ΔFX, and PPD3/Ad5ΔFX for their FX binding and subsequent neutralizing antibody binding.

